# Significant haematological alterations in clozapine-treated patients: prevalence and clinical correlation

**DOI:** 10.1017/neu.2024.54

**Published:** 2024-11-21

**Authors:** Muhammed Fatih Tabara, Cafer Baris Akar, Mehmet Kadir Atdagi, Mehmet Gurkan Gurok, Murad Atmaca

**Affiliations:** 1 Department of Psychiatry, Firat University School of Medicine, Elazig, Turkey; 2 Child and Adolescent Psychiatry, Firat University School of Medicine, Elazig, Turkey

**Keywords:** Clozapine, haematological adverse effects, leucocytosis, red blood cell distribution width, mean platelet volume, mean corpuscular haemoglobin concentration

## Abstract

**Objectives:**

Clozapine is an atypical antipsychotic crucial for treatment-resistant schizophrenia, characterised by its multi-receptor targeting, including serotonin (5-HT2A, 5-HT2C) and dopamine (D1, D2, D3, D4) receptors, among others. This broad mechanism is effective against positive symptoms of schizophrenia with a lower incidence of extrapyramidal side effects. However, clozapine poses significant haematological risks, notably agranulocytosis, necessitating stringent blood monitoring protocols.

**Methods:**

This study examined haematological parameters in 157 patients on clozapine therapy, analysing the prevalence and clinical correlations of haematological abnormalities such as leucocytosis, thrombocytosis, and alterations in red blood cell distribution width (RDW) and mean platelet volume (MPV).

**Results:**

The findings revealed leucocytosis in 36.9% of patients, thrombocytosis in 8.9%, and elevated RDW in 23.6%. Notably, higher clozapine doses were associated with leucocytosis, though no significant correlations were found between clozapine dose, duration of use, and changes in RDW, mean corpuscular haemoglobin concentration, or MPV.

**Conclusion:**

The study’s results underscore the necessity of regular haematological monitoring to mitigate the risks of clozapine therapy while leveraging its therapeutic benefits. Additionally, the study suggests personalised dosing strategies to balance efficacy and safety, particularly in managing clozapine-induced haematological changes.

## Significant outcomes

- Elevated RDW may indicate bone marrow suppression in clozapine-treated patients.
- Clozapine treatment led to leucocytosis in 36.9% of the study’s patients.
- 65% of patients had increased MPV, suggesting clozapine impacts platelet turnover.

## Limitations

The study has several limitations. One of the major limitations is that the sample consisted of 157 patients. Because the design of the study is observational, it cannot establish causality between clozapine use and hematologic changes. The use of additional medications alongside clozapine in most patients introduces potential confounding variables that might influence haematological outcomes. Finally, the varying duration of clozapine use among patients (ranging from 1 to 124 months) could affect the consistency of results and their interpretation.

## Introduction

Clozapine is an atypical antipsychotic medication primarily used to treat treatment-resistant schizophrenia. In neuroscience-based nomenclature, clozapine is classified according to its pharmacological profile and receptor targets. Clozapine is a multi-acting receptor-targeted antipsychotic. This classification underscores its broad mechanism of action, involving multiple neurotransmitter systems (Zohar *et al*., 2015). Clozapine exhibits high affinity for several serotonin receptors (5-HT2A, 5-HT2C, 5-HT6, and 5-HT7), dopamine receptors (D1, D2, D3, and D4), and other receptors such as histamine H1, adrenergic α1 and α2, and muscarinic acetylcholine receptors (M1–M5). Its antagonism at the 5-HT2A receptor is particularly significant, as this action is thought to contribute to its antipsychotic effects and lower incidence of extrapyramidal adverse effects compared to typical antipsychotics. The blockade of D2 receptors, while moderate, is crucial in mitigating the positive symptoms of schizophrenia, such as hallucinations and delusions. Clozapine’s effects on the muscarinic and adrenergic receptors account for some of its adverse effects, including sedation and orthostatic hypotension (Stahl, 2021). This complex receptor interaction profile of clozapine not only highlights its effectiveness in treatment-resistant schizophrenia but also explains its unique adverse effect profile, necessitating careful monitoring and management of patients.

Clozapine is associated with several significant adverse effects, among which haematological concerns, particularly agranulocytosis, are the most serious. Agranulocytosis is a potentially life-threatening condition characterised by an extremely low count of neutrophils, a type of white blood cell (WBC) essential for combating infections (Magistri & Mellini, 2023). It is still unclear the mechanism by which clozapine causes agranulocytosis. In general, it is thought that there may be a genetic predisposition, but clozapine or its metabolites may cause agranulocytosis through an immune-mediated response (Sernoskie *et al*., 2023). There are also experimental studies showing that clozapine and its metabolites have a direct toxic effect on bone marrow (Lahdelma *et al*., 2010). This adverse effect necessitates stringent blood monitoring protocols to ensure patient safety. Regular blood tests are mandated weekly for the first six months of clozapine therapy, bi-weekly for the next six months, and monthly thereafter (US Food & Drug Administration, 2023). This monitoring helps detect early signs of neutropenia (a precursor to agranulocytosis), allowing for prompt intervention before severe complications arise. While this rigorous monitoring can be burdensome, it is essential for mitigating the risks associated with clozapine, ensuring that its therapeutic benefits for treatment-resistant schizophrenia outweigh the potential dangers. Besides agranulocytosis, clozapine can have other effects on blood parameters. Clozapine can cause an increase in WBC count, which is known as leucocytosis. The aetiology and risk factors of clozapine-induced leukocytosis remain unclear. Clozapine-induced leukocytosis may result from the stimulation of cytokines like TNFα, IL-2, IL-6, and G-CSF. These cytokines prevent apoptosis by upregulating anti-apoptotic proteins and promote the maturation of myeloid cells into granulocytes, leading to increased leukocyte levels (Fehsel *et al*., 2005). Clozapine-induced leucocytosis is usually mild and transient. (Paribello *et al*., 2021). Clozapine can also cause a general reduction in white blood cells (leukopenia), though it is typically less severe than agranulocytosis (Copolov *et al*., 1998). This broader decrease can still impair the immune system and increase susceptibility to infections. In a comprehensive retrospective study, the incidence of leucopoenia in patients using clozapine was found to be 0.38%. In the same study, it was emphasised that clozapine was similar to other antipsychotics in terms of leukopoenia risk (Geldenhuys *et al*., 2021). Clozapine may lead to an elevation in platelet count, a condition called thrombocytosis. Like leucocytosis, this effect is generally mild and reversible (Hampson, 2000). In addition to thrombocytosis, there have been reports of thrombocytopenia in patients taking clozapine (Atmaca *et al*., 2013).

Red cell distribution width (RDW) is a measure of the variation in the size or volume of red blood cells (RBCs). It has been reported that elevated RDW values may be an indicator of nutritional deficiencies and inflammation and may also be an indicator of mortality in some cardiac diseases such as heart failure and myocardial infarction (Danese *et al*., 2015; Haenggi *et al*., 2024). RDW is also increased in cases of bone marrow suppression. It has been suggested that clozapine or its metabolites may cause hematologic adverse effects by suppressing bone marrow (Magistri & Mellini, 2023). From this point of view, changes in RDW values in patients taking clozapine may be a tool used to indicate adverse effects.

Clozapine is one of the most commonly used drugs by clinicians, especially in treatment-resistant psychotic cases. However, we believe that it is not used to the extent it deserves in practice with the assumption that it has serious hematologic adverse effects and should be closely monitored. In this study, we aimed to determine the frequency of changes in hematologic parameters by complete blood count and their relationship with clinical variables in patients who had been receiving various doses of clozapine treatment for some time.

## Methods

### Study desing and participants

This study was conducted to evaluate the haematological adverse effects associated with clozapine treatment in a sample of 157 patients. The participants were recruited from both outpatient and inpatient clinics at our medical centre, with the inclusion period spanning from January 1, 2023, to April 1, 2024. Ethical approval for the study was obtained from the Ethics Committee of Firat University School of Medicine. Written informed consent was obtained from all participants after the study procedures were explained. The procedures followed were in accordance with the Helsinki Declaration of 1975, as revised in 1983.

The inclusion criteria were that the participants were between the ages of 18–65 and had been on clozapine for at least 1 month. Exclusion criteria included having a medical illness other than psychiatric disorder, having recently used a drug or supplement that may affect blood parameters, having recently given blood or having received blood products, presence of a history of alcohol and/or substance use disorder in the past six months and refusal to give written consent.

Heparinised venous blood samples were taken from the patients’ forearm veins. Blood samples for each subject were collected in 5 ml vacutainer tubes containing potassium EDTA. Using an auto-analyser (Coulter Max M, Coulter Electronics Ltd, Luton, U.K.), all total blood count parameters were detected.

### Statistical analysis

All statistical evaluations were performed using version 22.0 of the Statistical Package for Social Sciences programme (SPSS for Windows, version 22.0, SPSS, Chicago, IL, USA). The Kolmogorov-Smirnov test was used to determine if the data displayed a normal distribution or not. Normally distributed continuous numerical data were evaluated by Student’s *t*-test. Categorical variables were compared with Chi-square test. The relationship between the variables was assessed using the Pearson correlation test. In all evaluations, *p* < 0.05 was accepted as the level of statistical significance.

## Results

The study included 157 patients, 65.6% (*n* = 103) of whom were male. The mean age of the patients was 38.28 ± 12.04 years. The mean illness duration of the patients was 12.89 ± 9.52 (min = 1, max = 40). The distribution of the diagnoses of the patients was as follows: 39.5% (*n* = 62) schizophrenia, 38.9% (*n* = 61) bipolar disorder, 12.1% (*n* = 19) schizoaffective disorder and 9.6% (*n* = 15) depression with psychotic features. Only 7 (4.5%) of the patients were followed up with clozapine monotherapy, while the others were receiving at least 1 additional drug. The most commonly used drug in addition to clozapine was sodium valproate with 24.2% (*n* = 38). The second most commonly used drug was lithium carbonate with 11.4% (*n* = 18) and the third most commonly used drug was amisulpride with 6.3% (*n* = 10). The mean duration of clozapine use was found to be 8.66 ± 22.57 (min = 1, max = 124) months. The mean clozapine dose was 202.87 ± 106.44 mg/day (min = 25, max = 700). When asked about suicide attempts, 29 patients had attempted suicide at least once.

Of the patients, 31.8% (*n* = 50) had an additional medical illness. When the most prevalent comorbidities were examined, it was observed that 26% *(n* = 13) of the patients had diabetes mellitus, 22% (*n* = 11) had hypertension, and 14% (*n* = 7) had hypothyroidism. The smoking rate of the patients was 51% (*n* = 80), while alcohol use was 8.3% (*n* = 13). Socio-demographic data of the patients are presented in Table 1.

**Table 1.** Sociodemografic data of participants

	Number (Percent)
Age (Mean±SD)	38.28 ± 12.04
Gender	
Male	103 (65.6%)
Female	54 (34.4%)
Smoking	
Yes	80 (51.0%)
No	77 (49.0%)
Alcohol use	
Yes	13 (8.3%)
No	144 (91.7%)
Diagnosis	
Schizophrenia	62 (39.5%)
Bipolar disorder	61 (38.9%)
Schizoaffective disorder	19 (12.1%)
Major depression with psychotic features	15 (9.6%)
Duration of illness (year)	12.89 ± 9.52
Clozapine dosage (mg/day)	202.87 ± 106.44
Duration of clozapine use (month)	8.66 ± 22.57
Additional medical illness	
Yes	50 (31.8%)
No	107 (68.2%)
Presence of suicide attempt	
Yes	29 (18.5%)
No	128 (81.5%)

Leucocytosis was found in 36.9% (*n* = 58) of the patients. Thrombocytosis was observed in 8.9% (*n* = 14) of patients and thrombocytopenia in 1.3% (*n* = 2). RBCs were below the normal range in 11.5% (*n* = 18) of the patients and above the normal range in 1.9% (*n* = 3). Mean corpuscular haemoglobin concentration (MCHC) levels were found to be above the normal range in 31.8% (*n* = 50) of the patients. Red blood cell distribution width (RDW) was found to be above the normal limit in 23.6% (*n* = 37) of the patients. Mean platelet volume (MPV) values were above the normal range in 65% (*n* = 102) of the patients. Complete blood count data of the patients are shown in Table 2.

**Table 2.** Haematologic parameters of the patients

		Patients (Number/Percent)	Reference values
WBC	<3.8 3.8-8.6 >8.6	0 99 (63.1%) 58 (36.9%)	3.8–8.6 x10^3^/µL
RBC	<4.1 4.1-6.0 >6.0	18 (11.5%) 136 (86.6%) 3 (1.9%)	4.1–6.0 x10^6^/ µL
HGB	<11.1 11.1-17.1 >17.1	12 (7.6%) 139 (88.5%) 6 (3.8%)	11.1–17.1 g/dL
HCT	<33 33-57 >57	6 (3.8%) 151 (96.2%) 0	33–57 %
MCV	<76 76-100 >100	7 (4.5%) 148 (94.3%) 2 (1.3%)	76–100 fL
MCH	<24 24-31 >31	10 (6.4%) 113 (72.0%) 34 (21.7%)	24–31 pg
MCHC	<28 28-34 >34	1 (0.6%) 106 (67.5%) 50 (31.8%)	28–34 g/dL
RDW	<12 12-15 >15	7 (4.5%) 113 (72.0%) 37 (23.6%)	12–15 %
PLT	<140 140-360 >360	2 (1.3%) 141 (89.8%) 14 (8.9%)	140–360 x10^3^/µL
MPV	<7 7-9 >9	3 (1.9%) 52 (33.1%) 102 (65.0%)	7–9 fL

WBC, White blood cell; RBC, Red blood cell; HGB, Hemoglobin; HCT, Haematocrit; MCV, Mean corpuscular volume; MCH, Mean corpuscular haemoglobin; MCHC, Mean corpuscular haemoglobin concentration; RDW, Red cell distrubition width; PLT, Platelets; MPV, Mean platelets volume.

Lithium carbonate and sodium valproate are known to have effects on blood counts, but not commonly. The effect of these two drugs on the positive findings obtained in the study was investigated and no statistically significant effect was found (*p* > 0.05).

When the relationship between hematologic changes and clozapine dose was examined, it was found that patients with leucocytosis used statistically significantly higher doses of clozapine, but clozapine doses were similar for RDW, MCHC and MPV changes. No significant correlation was found between the dose of clozapine, the duration of use, and the hematologic parameters.

## Discussion

The findings of this study offer important insights into the haematological effects of clozapine treatment in patients with schizophrenia and other psychiatric disorders. Our results highlight several key points that warrant further discussion and have significant clinical implications.

Firstly, the incidence of leukocytosis observed in 36.9% of patients is noteworthy. This aligns with previous reports suggesting that clozapine can cause an increase in white blood cell counts, an effect that is usually mild and transient (Fabrazzo *et al*., 2017; De Berardis *et al*., 2018). Our analysis demonstrated a statistically significant correlation between higher doses of clozapine and the presence of leukocytosis, indicating that dose management might be crucial in mitigating this side effect. Given the critical need for regular blood monitoring during clozapine therapy, this finding underscores the importance of dose adjustments and vigilant follow-up. In a recent study, the prevalence of leukocytosis in patients using clozapine was reported to be 37.8%. The same study also reported that lithium use and male gender were risk factors for leukocytosis (Fabrazzo *et al*., 2017). In our study, there was no difference in the prevalence of leukocytosis based on gender and lithium use. In a study conducted by Yang et al., four groups of patients were compared: clozapine only, lithium in addition to clozapine, valproate in addition to clozapine, and both lithium and valproate in addition to clozapine. Leucocytosis was reported to occur in 41.79% of all patients. It was also reported that male sex, young age, daily dose of clozapine, and concomitant use of lithium and benzodiazepines increased the risk of leukocytosis (Yang *et al*., 2023).

The elevated levels of MCHC found in approximately one-third of the patients suggest that clozapine may impact red blood cell parameters. This alteration in MCHC levels could be indicative of changes in red blood cell metabolism or bone marrow function, potentially linked to the drug’s complex pharmacological profile. While the clinical significance of this finding requires further investigation, it highlights the necessity of monitoring red blood cell indices as part of routine haematological assessments in patients undergoing clozapine treatment. The fact that there are no studies in the literature on the change in MCHC levels in patients taking clozapine makes our finding valuable.

Another critical finding was the increased red blood cell distribution width (RDW) in 23.6% of the patients. Elevated RDW is often associated with various clinical conditions, including nutritional deficiencies and bone marrow suppression (Danese *et al*., 2015; Haenggi *et al*., 2024). Given the suggestion that clozapine or its metabolites might induce bone marrow suppression, monitoring RDW could serve as an early indicator of adverse haematological effects, enabling timely intervention (Magistri & Mellini, 2023). In the study by Bouvier et al., comparing clozapine and haloperidol for adverse effects, it was reported that the RDW was significantly higher in male patients taking clozapine (Bouvier *et al*., 2022). Since RDW increase is an indicator of inflammation, it has been studied in the literature in various psychiatric illnesses (Najjar *et al*., 2013; Sathe *et al*., 2024). However, there are not enough studies showing its association with clozapine. Therefore, further studies are needed to demonstrate the reproducibility of the finding of increased RDW in patients taking clozapine.

The most striking result was the high prevalence (65%) of increased MPV, which indicates changes in platelet size. This finding suggests that clozapine might influence platelet production or turnover. Although thrombocytosis and thrombocytopenia were observed in fewer patients (8.9% and 1.3%, respectively), the significant proportion with elevated MPV warrants attention, as changes in platelet size can be associated with various cardiovascular risks and inflammatory states (Beyan *et al*., 2010; Korniluk *et al*., 2019). Regarding psychiatric disorders, there are studies reporting that the MPV value increases in patients with major depression and manic episode bipolar disorder, and decreases in patients with first-episode schizophrenia (Almış & Eğilmez, 2021; Fábián *et al*., 2022; Wei *et al*., 2022). There aren’t enough studies looking at how clozapine affects MPV. In a follow-up study conducted in schizophrenia patients, patients were followed up for 1 year after the initiation of clozapine treatment and it was found that there was no significant change in MPV values (Lee *et al*., 2014). Given its inflammatory and metabolic adverse effects, clozapine may have contributed to MPV increase; nevertheless, additional research is required to draw a firm conclusion.

Interestingly, despite these haematological changes, our study found no significant correlation between clozapine dose or duration of use and the majority of the haematological parameters examined. This lack of a dose-response relationship suggests that individual variability and possibly genetic predispositions might play a larger role in the haematological effects of clozapine than previously thought. Further research exploring genetic markers and individual patient characteristics could provide deeper insights into the mechanisms underlying these effects and help tailor personalised treatment plans.

We would like to conclude the discussion by mentioning which gaps in the literature this study fills and its clinical significance. The influence of clozapine on mean corpuscular haemoglobin concentration (MCHC) and red blood cell distribution width (RDW), two characteristics that are not well-explored in the context of clozapine treatment, is identified in the study as a substantial research need. For example, the study discovers that high RDW is present in 23.6% of patients, indicating a potential bone marrow suppression; this is an area that needs more research. Furthermore, elevated MCHC levels were seen in 31.8% of patients, a result that hasn’t been thoroughly reported in previous research. The incomplete knowledge of the relationship between clozapine and mean platelet volume (MPV) is another gap. In 65% of patients, the research found an increase in MPV, which may indicate alterations in platelet turnover and be related to the inflammatory or cardiovascular adverse effects of clozapine. The results of the study demonstrate the importance of routine blood count follow-up in patients on clozapine. This would make it possible to identify side effects including leucocytosis, inhibition of the bone marrow, and abnormalities in platelets early on and allow for prompt therapies. Because leucocytosis and higher clozapine dosages are correlated, clinicians should think about customised dosing plans to balance therapeutic advantages and lower haematological hazards. The findings of blood tests may need regular dosage changes. By keeping an eye on RDW, clinicians may be able to detect bone marrow suppression early and take action before more serious effects arise. Likewise, a rise in MPV should lead to further evaluations of cardiovascular risk, particularly in those with pre-existing risk factors.

## Conclusion

In conclusion, while clozapine remains a vital option for treatment-resistant schizophrenia, its use requires careful monitoring of haematological parameters due to the risk of significant side effects. Regular blood tests, including white blood cell counts, MCHC, RDW, and MPV, should be integral to the management of patients on clozapine. These measures will help mitigate potential adverse effects and ensure that the therapeutic benefits of clozapine can be safely realised. Further studies are needed to elucidate the mechanisms of these haematological changes and to develop strategies for predicting and managing these risks effectively.
